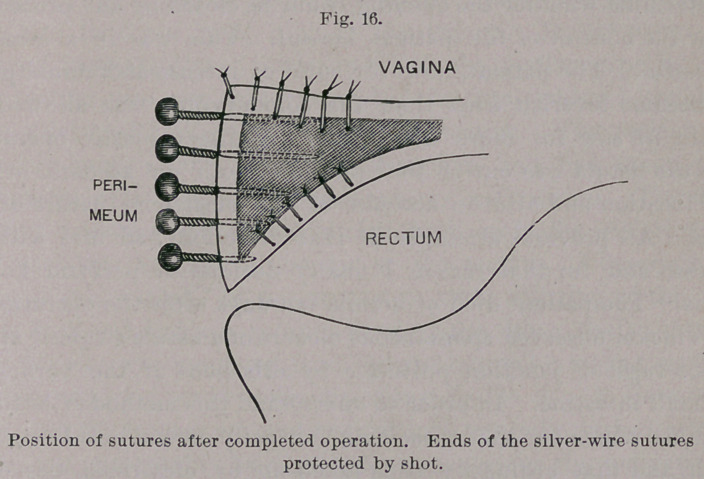# The Operative Treatment of Recto-Vaginal Fistula1In appreciation of the honor conferred by his election to Honorary Membership of the American Association of Obstetricians and Gynecologists. Reprinted from the Transactions, Vol. III., 1890, by permission of the author.

**Published:** 1891-06

**Authors:** Max Saenger

**Affiliations:** Leipsic, Germany


Buffalo Medical and Surgical Journal

VOL. XXX. JUNE, 1891. No. 11.

### Original Communications.

### THE OPERATIVE TREATMENT OF RECTO-VAGINAL FISTULA.[1]

BY DR. MAX SAENGER, Leipsic, Germany.

IN NO branch of operative gynecology is the number of methods so manifold as in the field of its plastic surgery. This may be, perhaps, explained by the present contest for supremacy between the operations of freshening and the flap-splitting methods. Indeed, one often hears that it is immaterial what method is used if success only follows. Nevertheless, that method is the best which concretely gives the best chance for primary union. But since in each instance only one operation can be selected, it becomes necessary to choose the one most fitting according to the conditions to be met. But the choice of a method for each special case, according to its special applicability, may be shown to rest often upon a paradoxical and unsafe basis. We expect more from plastic operations today than formerly—going, indeed, so far as to hope for invariable success.

Many gynecologists will agree in my experience that the eventual healing of these fistulæ is more difficult than is ordinarily supposed. When, for instance, Winckel expresses the opinion that they are more easily curable than urinary fistulæ, he must have in mind the more complicated cases of the latter condition, certainly not its more simple forms. Earlier in my experience I must confess to have had some failures in operations for recto-vaginal fistulæ, even in spite of the utmost care. The origin of the failure is clearly due to several possible causes—interference of or failure in the stitches to hold, or faulty approximation of the edges of the fistulæ. Again, the microörganisms in the rectum, or the presence of fecal matter or of flatus, may interfere with immediate union and cause suppuration. The aim of a perfect technique is, therefore, to

1. In appreciation of the honor conferred by his election to Honorary Membership of the American Association of Obstetricians and Gynecologists. Reprinted from the Transactions, Vol. III., 1890, by permission of the author.

obviate such interferences to success. The cases in which operation is required for the cure of recto-vaginal or perineal fistulæ are almost necessarily entirely of puerperal or traumatic origin. The para-rectal and para-vaginal abscesses giving rise to pus-producing fistulous channels emptying in the vagina and perineum, must be treated as *bona fide* fistulæ, either by slitting and curetting, or by the later method of cutting out the diseased tissues along their course and uniting their edges to secure primary union.

The elastic ligature may be considered obsolete. Fistulæ of the posterior vaginal wall, such as arise from pelvi-peritoneal abscesses, from ectopic pregnancy, and from suppurating cysts, particularly dermoids, and escaping into the rectum or vagina, must be classified apart from those now under consideration. The recto-vestibular variety of fistula, as also those generally found after failure of perineal plastic operations, must also be placed in a class of their own.

These preliminary observations will, perhaps, serve for the better appreciation of my special theme—to-wit, the operative treatment of typical fistulæ of the recto-vaginal septum. To these alone are the following conclusions to be applied. The operation for recto-perineal fistula, recto-laquearis, is especially to be mentioned. We now follow with a description and critique of the various methods :

1. Cauterization by chemicals ; the hot iron, thermo- or galvano-cautery, either from the vagina, or the rectum, or both. As long as the fistula has fresh granulating walls, cauterization, even in relatively extensive lesions, is able to accomplish much and give fair chance of healing. Nevertheless, opportunity does not often present itself to utilize the conditions necessary for success. In chronic fistulæ with cicatricial walls, cauterization will rarely be efficacious, unless, perhaps, in very small fistulous channels. It is to be recommended, therefore, that not only the fistulæ are to be cauterized, but also the tissue surrounding their opening into the vagina, to the extent of a half or one centimeter in diameter.

2. True surgical operations. These are to be made only from the vagina or the perineum. In some few cases Simon and Demarcquay have operated from the rectum in fistulæ located high up. Maurer attempted to close such fistulæ by transplantation of a rectal flap. That he met with little success is explained by my previous observations upon the causes of failure in these cases. We will see later that we possess in perineotomy a method by which we can reach a fistula of the vaginal roof from the perineum.

Trendelenburg now advocates a method similar to that followed out in the "sectio alta," by which the fistula is closed from the bladder, the patient having been put in the high pelvic position. I mention this comparatively, for while the bladder can be kept constantly drained, the same cannot be accomplished with the rectum. The true surgical operations may be divided into three classes:

I. Methods of denudation.
II. Methods of transplantation.
III. Methods of flap-splitting.

I. Denudation may be either (*a*) from the vagina, or (*b*) from the perineum after previous splitting of the fistula in the perineal bridge. The vagina denudation until recently generally conformed with that followed out in the Simon method of treating vesical fistulæ.

This consists in the removal of tissue along the edges of the fistula, usually a centimeter in width, while the sutures are introduced across its long diameter, both deep and superficial. (Figs. 1 and 2.)

Fig. 1.

Denudation of the fistulous edges in the vagina, with introduction of transverse sutures.

Fig. 2.

A. Freshened edges. S. Suture. F. Fistula.

The causes of failure in these operations have already been referred to. It is probable that in addition to such causes, carelessness in aseptic precautions, unsuitable and non-aseptic silk, or too soft catgut, must share the blame of failure.

By the use of silkworm-gut, silver wire, or even of fine aseptic silk, perfect results are to be expected in every case. Schauta (*Verhandlung d. Deutsch. Gesellsch. f. Gyn. zu München*, 1887,) made a notable improvement in this method; instead of freshening the edges of the fistula, he has brought it into a triangular form, corresponding to that used by Hegar in his method of posterior colporrhaphy. The fistula, therefore, lies in the center of a largely denuded surface and is closed by a larger number of stitches, which do not cross into the immediate territory of the fistula, whereby a much stronger primary union is attained. (Fig. 3.)

Fig. 3.

Triangular Denudation. (After Schauta.)

The simple denudation, as also the triangular, is not applicable in cases in which the bridge between the fistula is so narrow and thin that any further reduction of its extent will so reduce vascularity as to defeat the intent of the operation. Hitherto it has been the custom to cut through this remaining perineal bridge, and attempt to repair the injury by the triangular, butterfly-formed denudation of Hegar or Simon. These old methods were, for the most part, unsuitable to secure good results, because the point of defect usually lay high up in the recto-vaginal septum. Hence, not seldom did failure result, not only to close the original fistula, but often produced in addition others that did not before exist, from the rectum through the perineum. This, in most cases, was the fault of the method, not that of the operator.

Nevertheless some, Chrobak for instance, ("Über Mastdarmscheidenfisteln, nebst Bemerkungen über die Perinealnaht," *Wien. med. Blätter*, 1887, Nos. 27–33,) in spite of the defective methods, have reported excellent results, probably due to their excellent technique.

It will now be seen that however this operation is done, whether by the triangular denudation or by sutures from the vagina or perineum, (Baker Brown, Winckel, T. A. Emmet, Hirschberg, and others,) it is easily surpassed, both in safety and the certainty of results, by the flap-splitting operations—to which the future must afford still greater favor.

II. The transplantation of flaps from the vagina, the rectum, and the integument in a few cases has been attempted, so far as I am able to ascertain, with either entire or partial failure. Simon in one case had a favorable result by bringing a flap from the portio vaginalis into the fistula. Rydygier, Trendelenburg, and Leroy d'Etiolles succeeded in closing a urinary fistula by bringing flaps from the posterior vaginal wall.

It thus might be possible, indeed, to close fistulæ of the posterior wall by flaps from the anterior, the operation being simply reversed.

The methods of transplantation are only to be applied when the typical operations have failed, or have from the outset been impossible on account of the location of the fistula.

III. In the last few years I have chiefly used the flap-splitting method in closing recto-vaginal fistulæ. It is the intention of the present paper to present its claims for recognition as forcibly as possible.

It is well known that the method of Lawson Tait, long since applied, was the forerunner of a new era in perineorrhaphy. My attention having been called to this method of operating, I frequently discussed it in various papers, and further improved it. Since then the interest in this method of operating has become greater both in Germany and other countries, and the principles of the operation, both by Fritsch, von Herff, Walcher, and myself, have been transferred to the domain of operations for fistulæ. It is difficult today to understand why the principles of the flap-splitting methods are not more widely understood and appreciated, especially for fistulæ of the rectum, where every millimeter of tissue is precious. I hope now to be able to convince those who are still using the older methods, of the greater advantage and superiority of the flap-splitting operation. Here two methods are to be distinguished, according as the vagina or the perineum is taken as the point of departure. Each of these again has sub-divisions:

I. Flap-splitting operations from the vagina.

(*a*) Fritsch (*Centralbl. f. Gyn.*, Bd. xii. S. 804,) has invented an operation for small fistulæ, which is more a flap operation than a flap-splitting method, since the recto-vaginal septum is not separated into two leaves, but only a transverse, crescentic flap is taken from the vagina and drawn over the fistula, and there sutured. (V. Figs. 4 and 5.) *A B* is the crescentic incision through the vaginal mucous

Fig. 4.

Closure of fistula by transverse crescent-shaped lips. (After Fritsch.)

Fig. 5.

F. Fistula.

membrane along the upper margin of the fistula (F); moderate undermining of the flap to *C*. *A D B*, second deeper crescentic incision at a short distance from the fistula, adjoining with the removal of a crescentic portion of scar-tissue between both incisions. The so-formed upper convex flaps are then drawn over the fistula and sutured to the lower concave margin of the wound. Fritsch notes four successes following his method. I myself have tried it once and failed. The flaps failed to hold by the cutting through of some stitches, by which it happened that the contents of the bowel entered the covered but not closed fistula, and prevented healing. Fritsch pronounces his method unsuitable for larger fistulæ.

(*b*) The method of sagittal splitting of the recto-vaginal septum used by me is applicable to all kinds of rectal fistulæ in which it is possible to obtain vascular flaps from the vaginal wall. The higher the fistula is above the perineum the more suitable is the operation. The steps of the operation are two,—to-wit, the splitting of the septum and the suture. It will be most to the point to describe a typical operation (see Figs. 6 and 9). Frau B., *æt.* thirty-four years, had a complete tear of the perineum at her first confinement ten years ago. This was repaired at once. The perineum healed, but the torn recto-vaginal septum did not, and a large recto-vaginal fistula remained. Two attempts had been made to close it by the old Simon operation without success. There was present a normal-looking perineum of two and a half centimeters in depth, with competent sphincter ani. Two centimeters above the vaginal border was located a fistula of sufficient size to admit the passage of the little finger.

The tissue remaining between the fistula and the perineum was sufficient to permit the intra-vaginal operation, which was accordingly done after the most thorough antiseptic preparation—comprising both vaginal and external disinfection.

The left index finger was introduced into the rectum and the field of operation extended by the introduction of several forceps à Cremailliére. An incision was made both above and below the fistula

Fig. 4.

A B and C D, sagitto-median incision through the vagina. A E D and A F D, borders of the undermining of crescentic vaginal flaps.

Fig. 7.

Vaginal flaps separated and turned back. At the bottom, across the fistula, Lauenstein's sutures are applied, as in 1, 1, Fig. 8.

1.5 cm. long, in the median line, up to the rectum, together with coincident freeing of the vaginal flaps, which were extended universally about the fistula, the mucous membrane being undermined to a depth of from 1.2 to 1.4 cm.

After retraction of the edges of the flaps the fistula lay in the midst of an extensive rhomboidal denudation. Exsection of the scar tissue was not necessary. The steps by which closure was accomplished are now to be described.

The fistula itself was closed, from the wound outward, by eight fine silk sutures introduced after Lauenstein's method for plastic perineal operation,[1] and so that each edge of the wound pierced twice, similar to Lembert's intestinal suture.

After clipping away the ends of the threads the vaginal flaps we closed over the sutured fistula by deep silkworm-gut and superficial silk sutures. There were, therefore, three rows of sutures: one buried and two closed from the vagina.

Fig. 8.

1, 1. Buried suture of the rectal surface of fistula. 2, 2. Deep vaginal sutures. 3, 3. Superficial vaginal sutures. 4, 4. Rectal supportive sutures.

The idea now occurred to me to introduce sutures *from the rectal side*, and so bring together, over the site of the fistula, the folds of the rectal mucous membrane, by which fecal matter would be kept from interfering with the union of the flaps. The patient was accordingly placed in the Trendelenburg position—the rectum dilated and the site of the fistula exposed—after which the introduction of the sutures was easily accomplished. The mucous membrane of the bowel was stitched over the fistula without *any denudation whatever*, by the simple linear perforation of the tissues on either side of the fistula, and the subsequent knotting of the sutures.

The fistula was thus covered over. The ends of the sutures were then cut off close. I am accustomed in all plastic operations to close the wound so carefully and exactly that its edges can scarcely be determined. By the application of iodoform and packing with iodoform gauze twice or thrice daily in the vulva—

1. *Centralbl. für Gynäkol.*, Bd. x. S. 49.

after each catheterization—the wound remained dry and healed perfectly. The patient took twice daily, for six days, twenty drops tr. opii. The bowels were moved every two days. The wound was fully healed at the end of sixteen days.

Fig 9.

Closure of the vaginal incision by deep sutures, seen from above.

The rectal stitches had not been seen up to this time, but in all probability were cast off later. I ask whether a more logical or simple solution of the requirement presented than the above-described flap operation ? I wish to call especial attention here to my method of applying the sutures.

1. *I lay the greatest stress upon the suture introduced and buried beneath the flaps.* The simple suture is not here practicable since the stitch holes easily lead into the rectum and do not inclose sufficient tissue. Only when the fistula is exactly closed will the vaginal flaps, like the side doors of an altar shrine, exactly approximate in the middle line, thus restoring the natural form. I must also say[1] that *fine aseptic silk in sufficient quantity can be buried in the wound*, a fact of great importance in operations for the cure of fistulæ. The silk occupies less space, is thinner, does not swell like catgut, and yet is certainly absorbed after a long time, which, again, is an important consideration. The above-described *protective intestinal suture* has only been used by me in one case. I

1. I use only the Chinese silk, never the plaited silk, whose interstices are pockets for microbes. I treat my silk by boiling in a five per cent. solution of carbolic acid, and preserve it in a sublimate solution of 1:500.

believe it to be, however, so rational that I expect to use it in all future cases.

2. *Methods of operation for intestinal fistulæ by flap-splitting from the perineum.*

*a.* Without sagittal splitting of the perineo-vaginal bridge.

*b.* By previous splitting of the same. In the first division, *a*,

Fig. 10.

Vaginal incision closed. Introduction of rectal sutures.

we proceed exactly as in flap perineorrhaphy for incomplete rupture of the perineum (second and third grades), in which, according to the operator's judgment, the incision must be either ⊔, ∨ or ⌣-shaped, by which the vaginal flaps separated from the rectum are formed corresponding to my earlier description. V. Samml. klin. Vorträge von Volkmann, No. 301; *Centralbl. f. Gynäkol.*, 1889, No. 47; Verhandlung d. deutsch. Gesel. f. Gyn. in Freiburg, 1889. Compare also the paper of Werder, in the Transactions of the American Association of Obstetricians and Gynecologists, 1889.

It is evident, therefore, that both the bowel and the flaps are perforated by the fistula. In two cases of smaller fistulæ I paid no attention to these openings and performed the transverse perineal operation, taking care, nevertheless, that one of the silver sutures was passed behind the rectal fistula. In one case success followed, but in the second I found later a small recto-perineal fistula, which, since it was located close to the anus, and only at times allowed the

Fig. 11.

Simple perineal flap. A. Anus. 1, 2, 3, 4. Buried Lauenstein's sutures for closure of the rectal fistula. 5, 6, 7, 8. Perineal sutures. 9, 10, 11. Vaginal sutures to close openings in the vaginal flaps.

escape of thin feces, had never been noticed by the patient. The simple splitting of the fistulous tract into the rectum would result in cure, which this method is especially legitimate for recto-perineal fistulæ. Moreover, I close also here *recto-perineal fistulæ* from the wound out by several buried sutures, à la Lembert-Lauenstein. The perforation in the vaginal flaps can be eliminated very easily by fine silk sutures. Perfect union results. (*Vide* Fig 11.) This extremely simple method can be applied equally well both to small and deep-seated fistulæ as well as to vestibular fistulæ in relatively thick perinea with a functionally perfect sphincter. Under *b* the fistula is larger and situated higher, and consists, in addition, of only a skin perineal fragment, with an open, relaxed anus and an insufficient sphincter, from all of which *the necessity of splitting the perineal bridge is evident. With the exception of flap-*

Fig. 12.

Fistula with only perineal fragment remaining.

Fig. 13.

Perineo-vaginal bridge torn through. R. P. Posterior wall of rectum.

Fig. 14.

Diagrammatic representation of operation. R. L. Rectal flaps with sutures of Lauenstein. V. L. Vaginal flaps.

*perineorrhaphy this is entirely analogous to complete tear of the perineum into the rectum;* if this does not extend very high, the typical perineal operation with deep perineal sutures of silver wire and superficial vaginal, with several external rectal stitches of fine silk, will suffice. If the defect extends higher up to the greater calibre of the bowel, the tear must be closed by buried silk sutures

Fig. 15.

Anterior rectal wall closed by Lauenstein's deep sutures. P. N. Perineal sutures. V. N. Vaginal sutures.

after the manner of Lauenstein—from the wound outward. In other cases there is nothing to hinder the primary closing of the intestinal tear. (*Vide* Figs. 12 to 15.) In this manner perfect

Fig. 16.

Position of sutures after completed operation. Ends of the silver-wire sutures protected by shot.

union results, while by the old methods, by the more difficult triangular denudation, the results were never certain. In twenty cases of flap-splitting perineorrhaphy in complete tears of the perineum, with and without rectal fistula, I have never had to do a secondary operation. In those horrible cases, fortunately seldom met with, of *multiple fistulæ of the bladder and rectum toward the vagina*, it is evident that no hard and fast rules for operation can be laid down. Under such circumstances the experience, ingenuity, and patience of the operator must determine the measure of his success.

Also in high-lying *fistulæ of the posterior vaginal roof*, the same may be said. Up to the present time our operative resources were limited, but now it is to be hoped that by the present advanced technique a great change may be wrought. I myself have had but *one* such puzzling case during five years. An enormous pelvi-peritoneal exudate concomitantly penetrated both rectum and vagina.

There remained in the posterior vaginal roof a fistulous opening the size of the thumb nail, surrounded by a hard cicatricial margin, communicating with the rectum. Above Douglas's cul-de-sac the vagina was roofed over by fibrous masses. The entire contents of the bowel were discharged through the vagina.

Before the patient came into my hands she had been treated several years. Her general condition was poor, and she suffered from chronic nephritis. I first attempted to tampon the fistula by iodoform gauze and afterward by inserting a colpeurynter into the vagina with a view of softening the cicatricial tissue, as well as to determine whether the opening could be closed by any procedure under the control of the patient herself. This was in a measure successful. The patient, however, suffered intense abdominal pain, and became feverish each time the colpeurynter was allowed to remain *in situ* for more than a day. A direct plastic operation upon the fistula was out of the question, both on account of its high location and also on account of the cicatricial nature of its edges. All previous attempts in that direction had met with no success, and for that reason I finally decided to perform kolpo-kleisis. The patient died of uremia ten days after the operation.

Winckel observed spontaneous closure of a fistula situated at the cervico-vaginal junction posterior by adhesions of the border of the small intestine. In order to understand this method of healing, one must remember that these fistulæ usually extend into Douglas's pouch, and that ordinarily it is taken up by cicatricial tissue or exudates around the perforation, so that it is rare for a case to occur in which the small intestine can come into relation with the fistula.

In a further communication of Chrobak's, a fistula was closed by the adhesions of a retroflexed uterus, but this manner of closure is so rare that it cannot be depended upon; nevertheless the case suggests the method to be followed out when operation is resorted to,—to-wit, drawing down the uterus to fill up the fistulous opening; and when the organ is already retroverted, its suture in this position can offer no great difficulty. When the fistula is the result of the breaking through of a suppurating ectopic sac or of a dermoid cyst, these growths should be removed by abdominal section, and their sacs stitched to the external abdominal opening. This is rendered more easy by the high pelvic position during operation. In order to prevent failure of closure by slipping of the stitches, it is necessary to make use of intra-abdominal tampons of gauze through the abdominal incision. Another method promises much for the future,—to-wit, perineotomy (*vide* my article, *Archiv. f. Gyn.*, Vol. xxxvii.); it is done in the following manner: First there is transverse splitting of the perineum, with complete separation of the vagina from the rectum if it is necessary, followed by partial cutting through of the levator ani. This is followed by the closure of the opening into the vagina through the drawing down of the portio-vaginalis. Subsequently the vaginal wound is sutured, the whole to be followed by after-treatment with iodoform gauze.

Latterly my observations have led me to operate not only for fistulæ of the posterior vaginal wall by transverse perineotomy, but also in those lying high in the rectum. In my paper cited above I have mentioned a case in which the matter expelled from the rectum was pus mingled with hair, from which I concluded that a dermoid had suppurated into the rectum. The patient came into my hands after a further discharge of hair had taken place. A careful examination corroborated my supposition. The fistula was of roseate shape, drawn inward, high up in the posterior vaginal roof on the anterior border of the rectum. I determined to remove the cyst-wall by perineotomy. After splitting of the pelvic floor from one ischiatic protuberance to the other, the vagina in a few minutes was separated from the rectum, and a wound was present into which I could easily insert my double fist, in spite of the fact that the levator ani was not cut into but only pushed aside. I now met remarkably hard tumorous masses in the subperitoneal pelvic space, so that further advance was impossible. After unsuccessful attempts to enter, the thin anterior wall of the rectum tore through about two centimeters in length. The tear was immediately closed by numerous fine stitches. This closure was found to be water-tight by repeated filling of the rectum with water. The recto-vaginal space was loosely packed with iodoform gauze and the perineal wound reduced by several deep silver sutures. The patient was kept under opium for six days. When castor oil was given she had thin evacuations through the perineal wound. This was repeated at intervals of five days, and numerous discharges followed during this period through the fistulous opening. After about five weeks the whole wound was entirely cicatrized without any remnant of the perineal fistula. From the circumstance that not a single silk suture was cast off, one can well come to the conclusion that only a small opening had been formed in the line of the suture, and that this afterward was closed by granulation and the reunion of the vagina and rectum.

Certain it is that the conditions here described are identical with those found when a perineotomy is done by entire separation of the vagina from the rectum. By this means it is practicable to reach the upper edge of the fistulous opening—and I am convinced that this is possible in most cases—and accordingly it must be very easy to close the wound from the surface presenting. The reason that the reconstructed vagina affords a better covering for the rectum, so that in case suture of the latter should partially give way, there would only remain a recto-perineal fistula, whose closure could be expected by granulation and cicatricial contraction.

With this I close my communication, with the consciousness that I have advanced many ideas which are suggestive of operative procedure on new lines. This remark I would not venture did I not believe that the point of view in the most advanced and latest discussions upon the subject, as in Pozzi's treatise upon gynecology, as well as that of Edward W. Jenks, in the *American System of Gynecology*, Vol. ii., justifies the expression.

---

REFLEX DYSPEPSIA.—Many troublesome dyspepsias in females are due to reflex action, and are associated either with menorrhagia, or with an irritable ovary; just as the vomiting of early pregnancy is due to an allied condition of the organs of generation. Counter-irritation over the tender ovary, with sulphate of magnesia as a purgative, and bromides to quiet the nervous system, frequently succeeds even in rebellious cases.—*Med. World.*

## Figures and Tables

**Fig. 1. f1:**
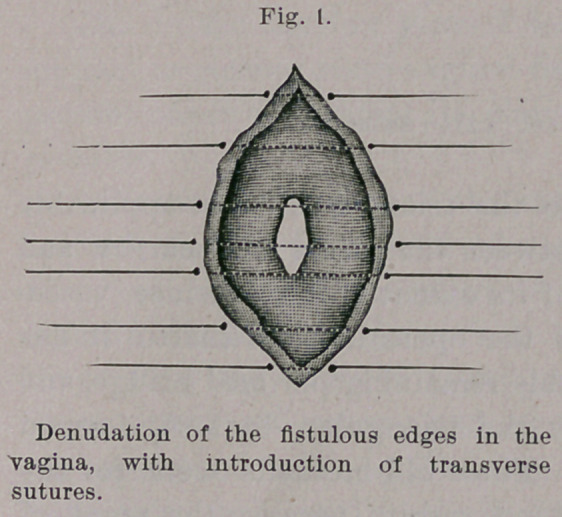


**Fig. 2. f2:**
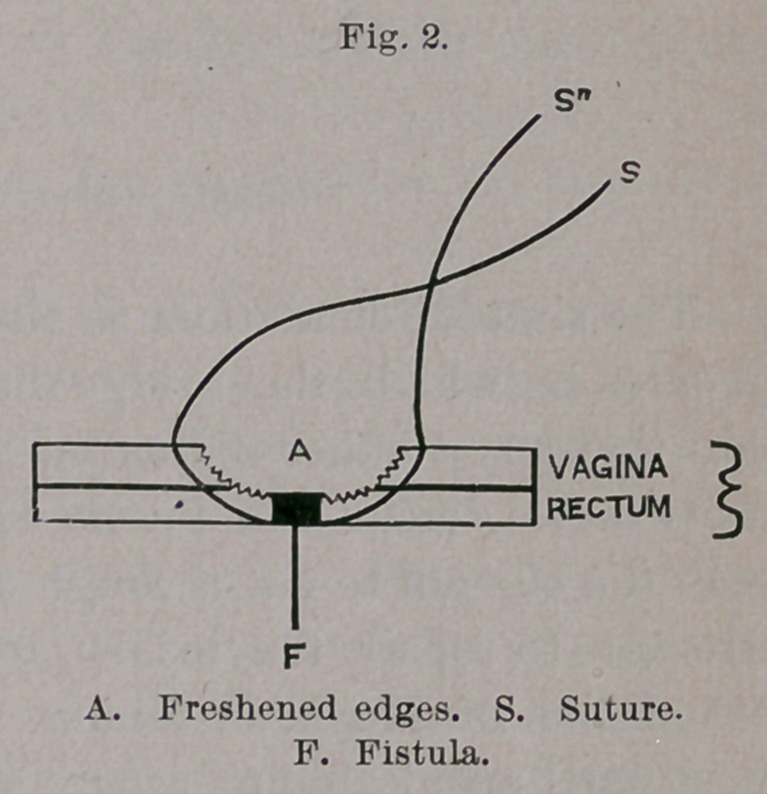


**Fig. 3. f3:**
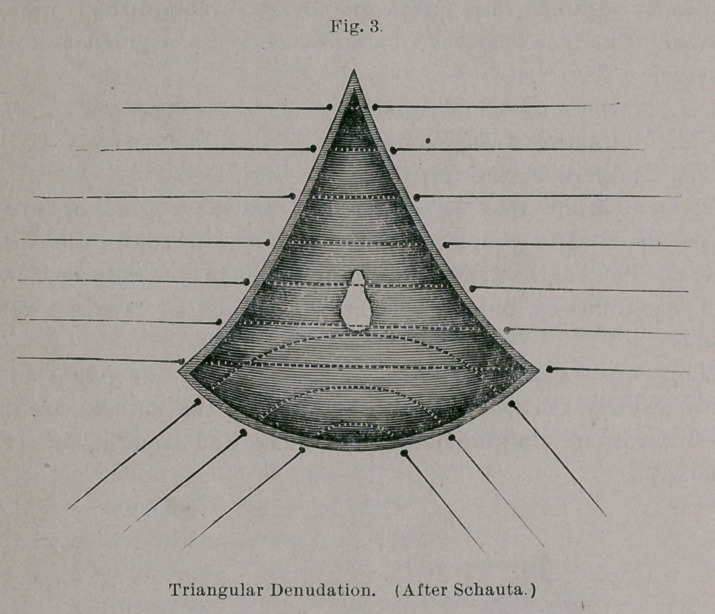


**Fig. 4. f4:**
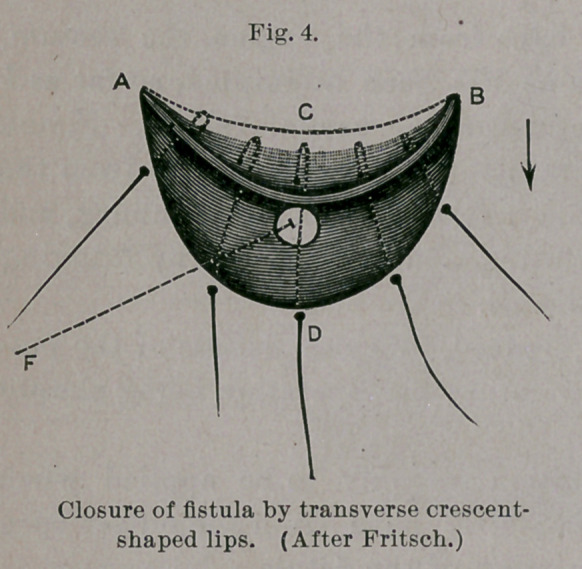


**Fig. 5. f5:**
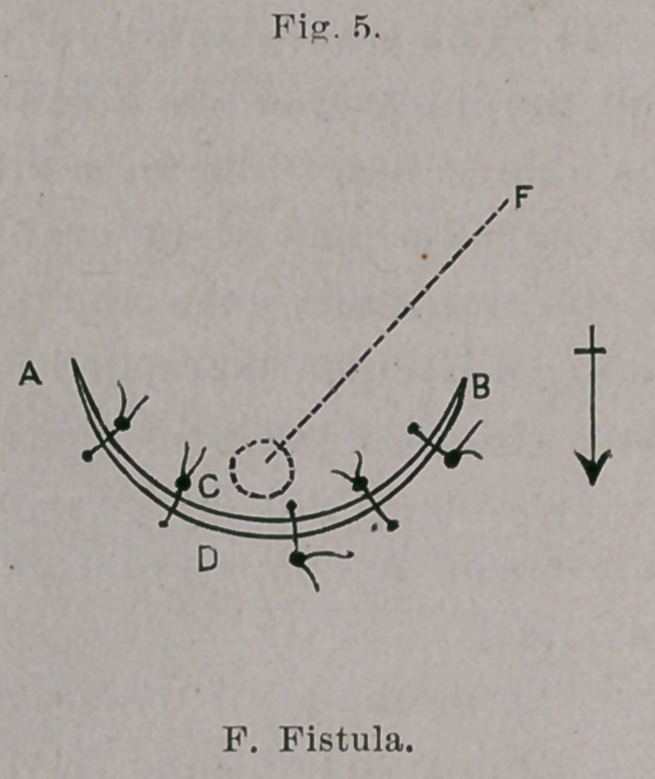


**Fig. 4. f6:**
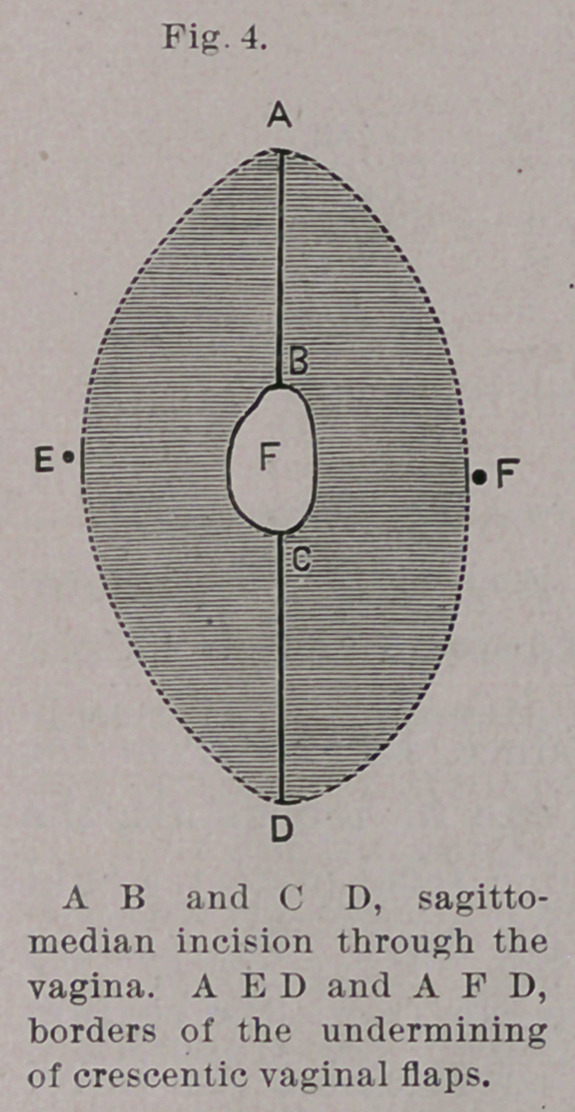


**Fig. 7. f7:**
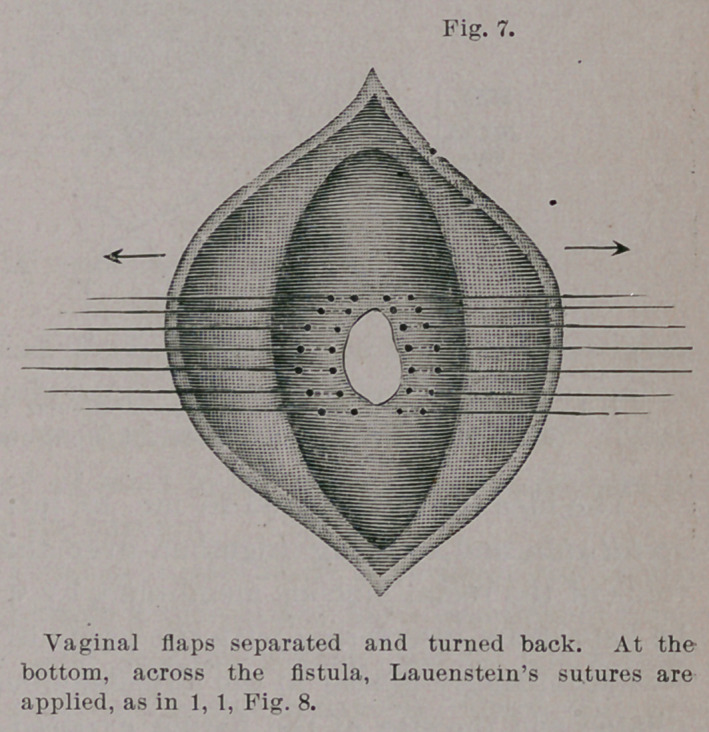


**Fig. 8. f8:**
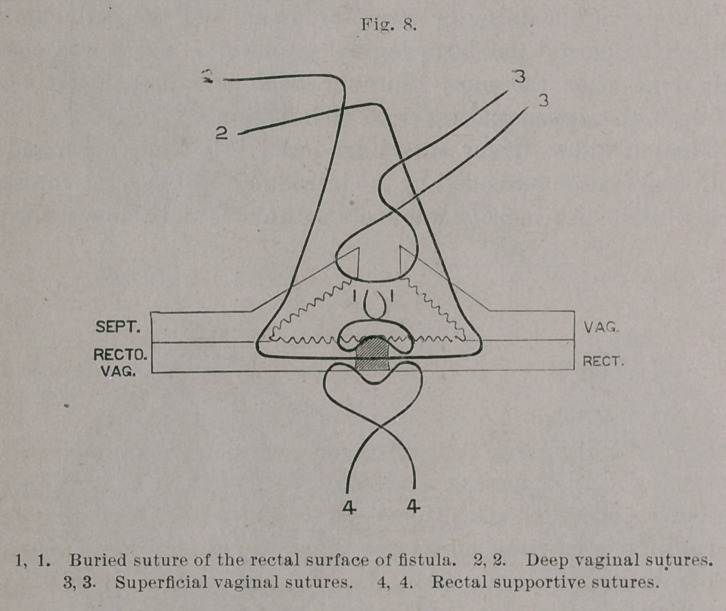


**Fig 9. f9:**
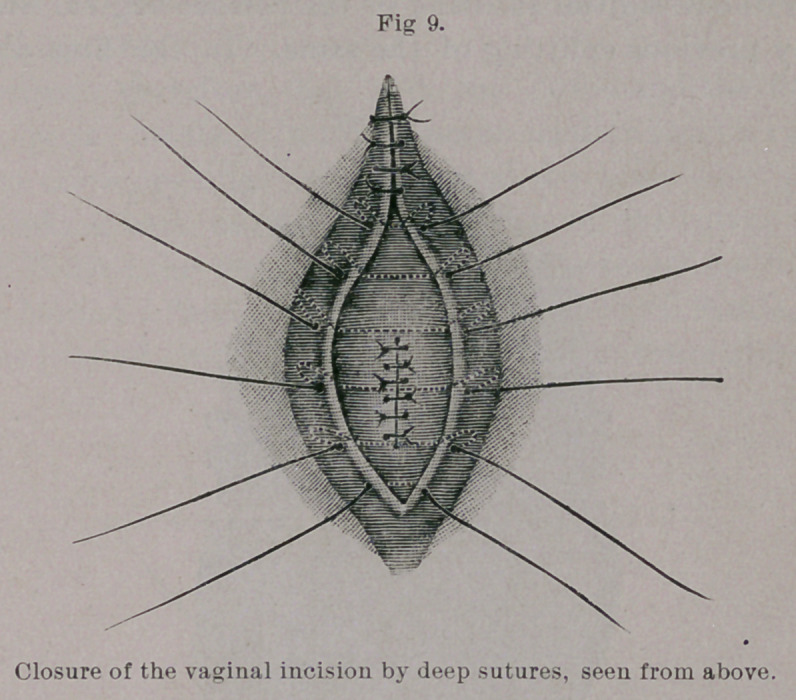


**Fig. 10. f10:**
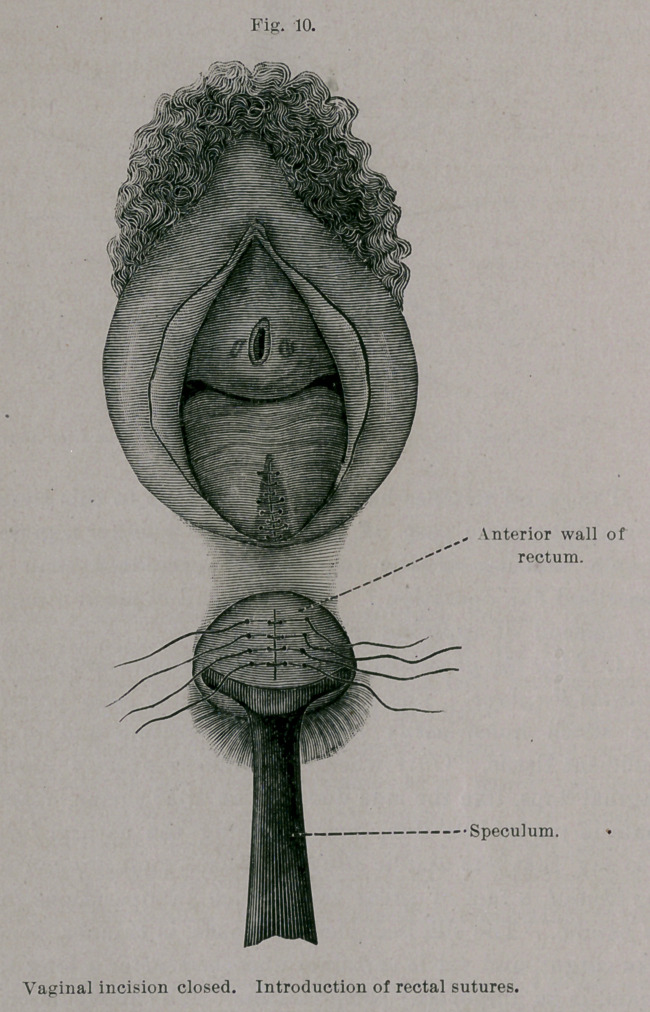


**Fig. 11. f11:**
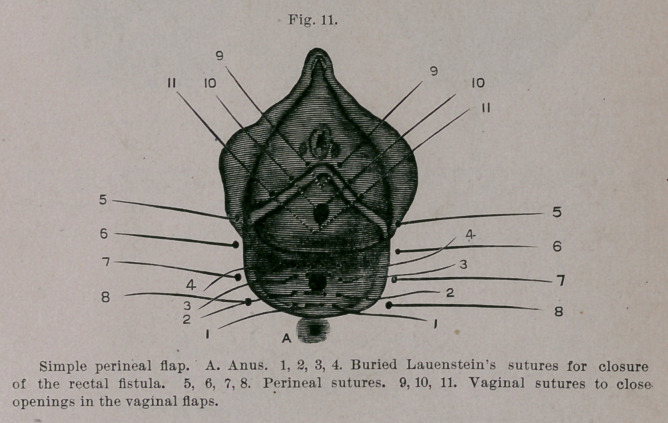


**Fig. 12. f12:**
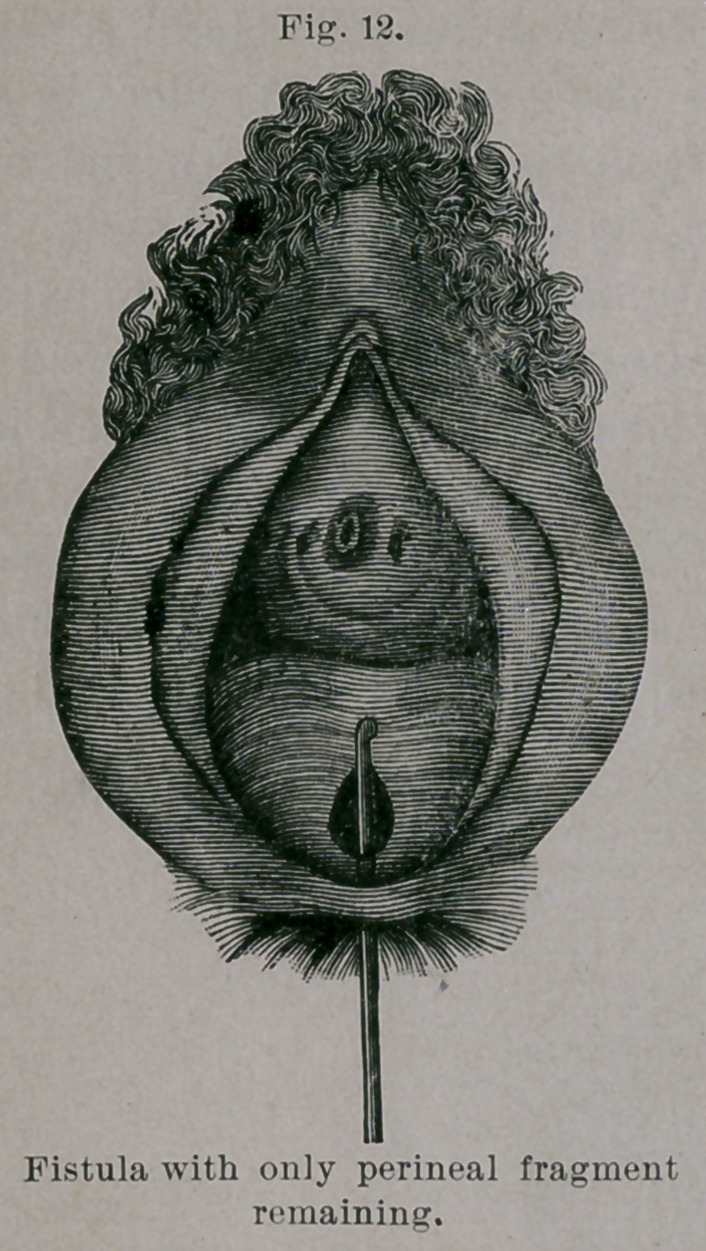


**Fig. 13. f13:**
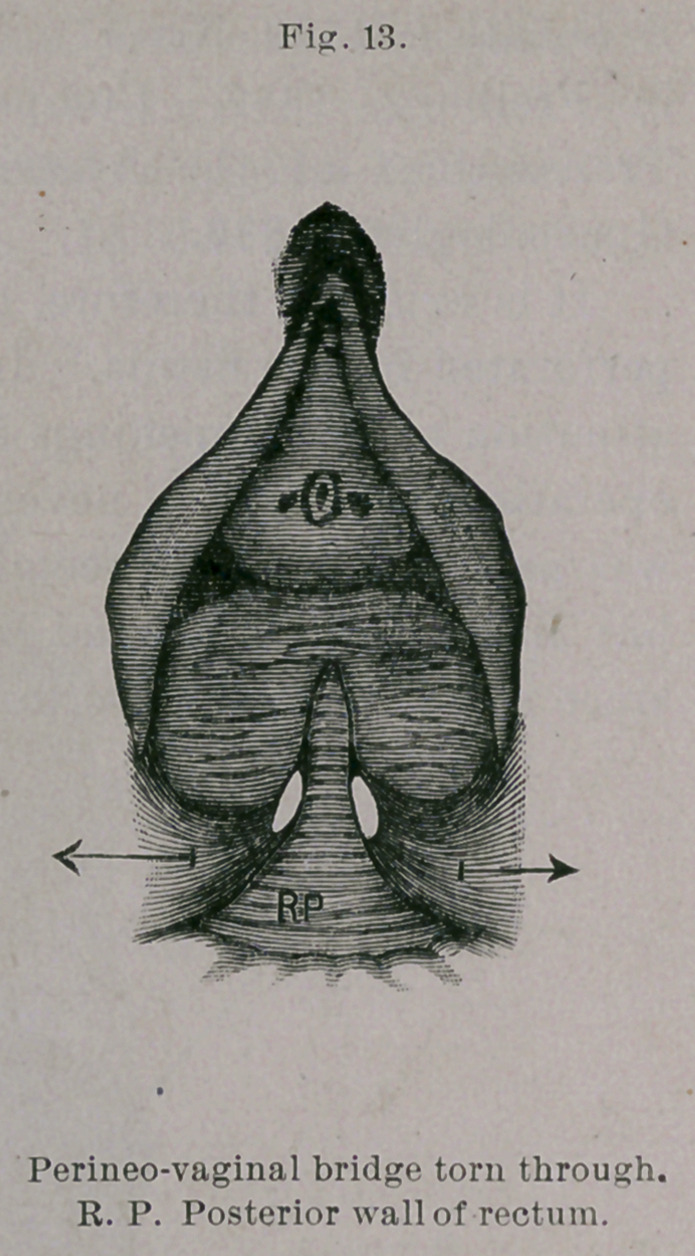


**Fig. 14. f14:**
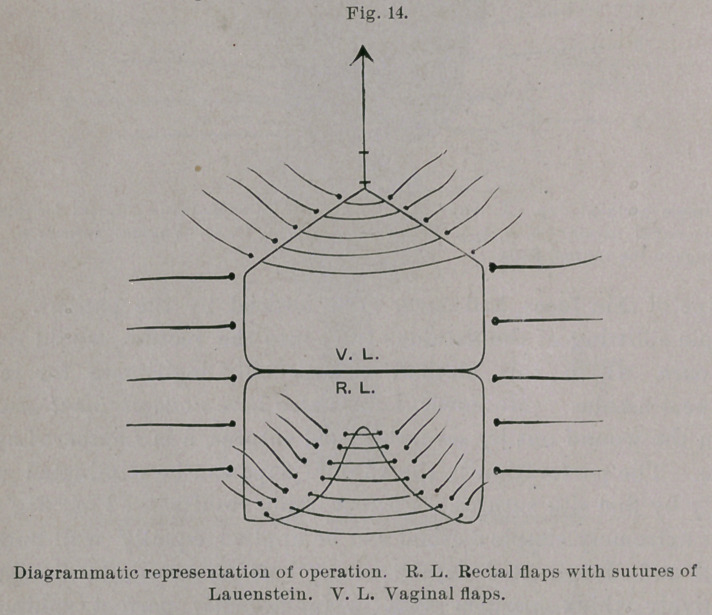


**Fig. 15. f15:**
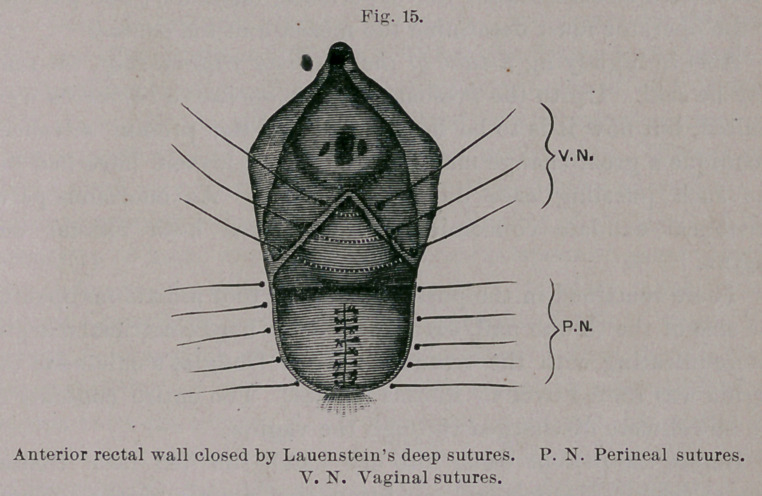


**Fig. 16. f16:**